# Case Report: Thigh anastomotic hemangioma

**DOI:** 10.3389/pore.2026.1612402

**Published:** 2026-04-14

**Authors:** Li Chen, Xia Gao, Qiang Zhang

**Affiliations:** 1 Department of Pathology, Deyang Hospital Affiliated Hospital of Chengdu University of Traditional Chinese Medicine, Deyang, Sichuan, China; 2 Department of Pathology, Pengzhou People’s Hospital, Chengdu, Sichuan, China; 3 Department of Pathology, Daying People’s Hospital, Suining, Sichuan, China

**Keywords:** anastomotic hemangioma, GNAQ, recurrence, the thigh, vascular tumor

## Abstract

Anastomotic hemangioma (AH) is a rare benign vascular tumor primarily occurring in the genitourinary tract; however, only two cases have been reported in the thigh. In this report, a 42-year-old female patient presented to the hospital for examination due to “a subcutaneous mass on the lateral aspect of the left thigh discovered 9 months ago, which has been gradually enlarging.” Subsequently, the lump was removed via local surgery. Histological examination reveals: At low magnification, the tumor was situated within the superficial subcutaneous fascia layer, presenting a loose lobular structure. Most of its margins were well - defined, while a small portion displayed expansile infiltrative changes. There were well - differentiated vascular lumens arranged in a communicating or anastomosing pattern, along with pseudopapillary structures. At high magnification, tumor cells were oval or short spindle - shaped, with vacuoles in the cytoplasm that contain red blood cells or homogeneously red - stained glassy globules. Moderate atypia was present, and mitotic activity was frequent, with hot spots averaging approximately 4/mm^2^. PCR-*GNAQ* mutation detection result: detected a missense mutation at codon 209 in exon 5 (*c.627A>T, p. Q209H*). Follow-up revealed tumor recurrence 10 months after surgery. Given the rarity of AH occurring on skin surfaces, coupled with the high proliferative activity observed in this case and its recurrence following excision, we report the diagnostic and therapeutic process along with the clinical and pathological features of this AH case. This aims to enhance the understanding of this disease among clinicians and pathologists.

## Introduction

AH is a rare benign vascular tumor characterized by well-defined borders, composed of thin-walled anastomosing vessels lined by a single layer of slightly protruding endothelial cells [1]. It was first described by Montgomery et al. [2] in the kidney. Due to its anastomotic or communicating - type vascular structure resembling splenic sinusoids, it can be mistaken for hemangiosarcoma.

## Case report

### Clinical history

A 42-year-old female presented with a subcutaneous mass on the lateral aspect of her left thigh, discovered 9 months prior and gradually enlarging. She reported no pain or itching. Physical examination revealed a well-defined mass approximately 2 cm in size, with no hyperpigmentation of the overlying skin. Surgical exploration showed the mass located subcutaneously on the lateral thigh, dark brown in color with a strawberry-like appearance. It was loosely adherent to surrounding tissues and easily dissected.

### Pathological examination

Macroscopic examination: A piece of skin-bearing tissue measured 2.5 cm × 2.0 cm × 1.0 cm, with a skin area of approximately 1.3 cm × 1.0 cm. Beneath the skin, there was a nodule that measured approximately 2.1 cm × 2.0 cm × 1.0 cm. Its cut surface was grayish-white to grayish-red, with a moderately firm consistency and visible hemorrhage.

**FIGURE 1:**
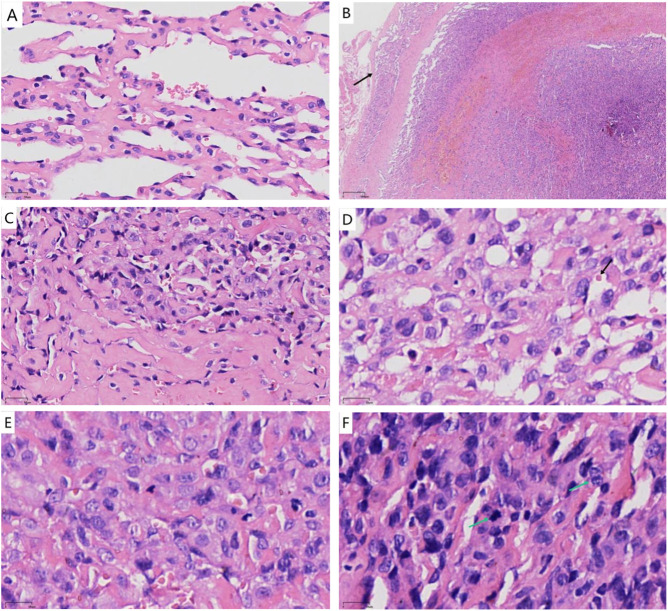
**(A)** The tumorous vascular lumen appears anastomotic. (H&E×200); **(B)** The tumor exhibits expansile infiltration locally, presenting anastomotic configuration, with some tumor cells growing in a solid pattern (H&E×20); **(C)** The stroma exhibited hyaline and mucoid degeneration of collagen (H&E×200); **(D)** Transparent small spheres can be observed in the cytoplasmic cavity (H&E×400); **(E)** Moderate atypia of cells in the solid tumor area (H&E×400); **(F)** Mitotic figures are easily observed. (H&E×400).

**FIGURE 2:**
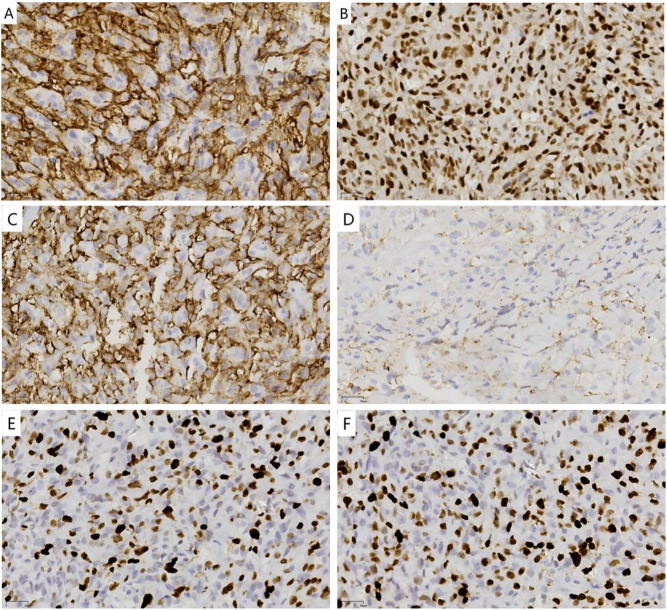
Immunohistochemical profile of the tumor. **(A)** Tumor cells show strong membranous staining for CD31. **(B)** Nuclear expression of Fli-1 is observed. **(C)** Tumor cells show strong membranous staining for CD34. **(D)** The cytoplasm of some tumor cells shows a relatively weak reaction to FVIII staining. **(E)**Expression of SMA in the nuclei of perivascular cells. **(F)** Ki-67 stain demonstrates a proliferation index of approximately 40%. (Envision, ×200).

Microscopic Features: At low magnification, the tumor was located within the superficial subcutaneous fascia layer, exhibiting a loose lobular structure with mostly well-defined borders. Well-differentiated vascular lumens were arranged in a communicating or anastomosing pattern (Figure 1A), and pseudopapillary structures were also visible. A small portion showed expansile infiltrative changes accompanied by hemorrhagic necrosis in some areas (Figure 1B). Surrounding cells were sparse. The lining cells were flat or boot-shaped, with a central region rich in cells arranged solidly and interspersed with small clefts lined by tumor cells. The stroma exhibited hyaline and mucoid degeneration of collagen (Figure 1C). Numerous hemorrhages and fibrinous thrombi were visible within the tumor lumens. Under high magnification, the tumor cells appeared oval or short spindle-shaped with indistinct borders. Cytoplasm was moderately abundant, eosinophilic, and contains vacuoles. Within these vacuoles, red blood cells or homogeneous red-stained glassy globules were visible (Figure 1D). And nuclei were round or oval, enlarged and deeply stained, exhibiting moderate atypia. The nuclear membrane was distinct yet slightly irregular, with moderately coarse chromatin and prominent nucleoli (Figure 1E). Mitotic figures were frequently observed, with approximately 4/mm^2^ in hot spots (Figure 1F). Immunohistochemistry: in tumor cells, CD31 (+) (Figure 2A), CD34 (+), Fli-1 (+) (Figure 2B), F-VIII (partially +), STAT-6 (−), p53 (wild-type expression), C-MYC (+, weakly, approximately 20%), S100 (−), EMA (−), D2-40 (−), Ki67(MXR002) (+, approximately 40%) (Figure 2C), SMA (+,perivascular cells). Preliminary diagnosis suggests a benign or borderline hemangioendothelioma. Hemorrhagic necrosis was visible in some areas, mitotic activity was prominent, and there was localized expansile infiltration. A well-differentiated angiosarcoma could not be ruled out. A specialized soft tissue pathology consultation at a tertiary hospital was recommended. Following consultation by soft tissue pathology specialists at West China Hospital of Sichuan University, the pathology report states: A highly vascular tumor with anastomotic features was observed. Differential diagnoses primarily included special types of hemangiomas and invasive vascular tumors (particularly well-differentiated angiosarcoma). Based on immunohistochemistry and genetic testing results, the findings were consistent with anastomotic hemangioma. Focal cellular proliferation was highly active. If complete resection had been achieved, close follow-up observation was recommended. Immunohistochemistry results: Vascular endothelial cells showed CD31 (+), CD34 (+), ERG (+), GLUT-1 (−), PHH3 (+), HHV-8 (−), p53 (focal+), ATRX (no significant loss), D2-40 (−), Ki-67 (MIB-1) (+, 20%, in hotspot areas); vascular wall showed SMA (+), Desmin (+).

Molecular pathology: PCR-*GNAQ* mutation detection identified a missense mutation at codon 209 in exon 5(*c.627A>T, p. Q209H*); PCR-*GNA11/GNA14* mutation detection did not detect hotspot mutations; PCR-*IDH1 (codon 132)/IDH2* mutation detection (codons 140/172) did not detect hotspot mutations. The sequencing was performed using the NGS platform provided by BGI, Chongqing, China.

### Treatment and follow-up

Recurrence occurred 10 months after surgical excision.

## Discussion

Diagnostic Features and Clinicopathologic Correlation: AH is a benign vascular tumor with unique morphology formally included in the WHO’s fifth edition of the classification of bone and soft tissue tumors. Hoxhaj I et al. [3] analyzed 159 cases of AH and found: ① The age of onset ranged from 15 to 85 years, with a median age of 58 years. There was no significant difference in incidence between males and females. Most cases were asymptomatic clinically, but some reported a history of renal impairment, hypertension, and malignant tumors; ② AH primarily occurred in the genitourinary tract, particularly the kidneys, adrenal glands, ovaries, and testes; ③ Beyond the genitourinary system, the most commonly affected organ was the liver, followed by paravertebral spaces, mediastinum, retroperitoneum, gastrointestinal tract, mammary glands, pericardium, and umbilical cord. In two reported cases, AH occurred in the thigh region [4, 5]: One case presented with multiple rashes on the left thigh, while the other exhibited leg pain.

Diagnosis and Differential Diagnosis: Under low-power microscopy, the tumor exhibits a loose, lobulated structure, often accompanied by local infiltration of adjacent tissues. It consists of interconnecting sinusoidal capillaries with visible intravascular thrombi and occasional extramedullary hematopoiesis. The lining cells are partially flattened and partially shoehorn-shaped, with lymphocytic infiltration possible in the stroma. Cellular atypia is mild to moderate; no severe atypia is present. Mitotic figures are absent or extremely rare [1]. However, in this case, the tumor cells exhibit moderate atypia with readily visible mitotic figures, representing an exceptional case. Intravascular thrombosis is a relatively typical phenomenon, and the presence of central sclerotic areas and localized necrotic foci is also a common feature [6]. Extramedullary hematopoiesis may be a prominent feature, with intravascular red-stained globules observed in certain cases [7]. Immunohistochemistry revealed positive staining for endothelial markers including CD34, CD31, Fli-1, and ERG. The vast majority of AH cases harbor activating mutations in the *GNAQ* or *GNA14* genes [8]. These genetic alterations are primarily observed in benign vascular tumors and have not been reported in malignant vascular tumors [9]. In this study, apart from its rare location and locally active cellular proliferation, the morphology, immunophenotype, and genetic mutations of AH were consistent with those reported in other literature.

Based on the location and morphological characteristics, AH in this report must be differentiated from the following diseases. ① Well-differentiated angiosarcoma: It may exhibit interconnected vessels and primitive blood cavities, with positive staining for vascular markers such as CD31 and Fli-1. However, well-differentiated angiosarcoma features cellular atypia and readily visible mitotic figures. In this study, the tumor exhibited expansile infiltrative growth locally, with areas showing diffuse solid cellular arrangement. Cellular morphology demonstrated moderate atypia and highly active mitotic figures. Therefore, in such cases, morphological features alone are insufficient to differentiate between the two. Highly differentiated angiosarcoma exhibits genetic alterations including *MYC* and *FLT* gene amplification and *TP53* gene mutations, while lacking *GNAQ*, *GNA14*, and *GNA11* mutations. In contrast, AH may harbor *GNAQ*, *GNA14*, and *GNA11* mutations. These genetic mutations serve as critical diagnostic criteria for differential diagnosis. ② Kaposi’s sarcoma: It affects the skin or lymph nodes of immunocompromised elderly patients, with prominent spindle cells in the tumor components. In this case, the patient’s medical history revealed no immunodeficiency-related diseases, and the absence of diagnostic HHV-8 positivity excludes Kaposi’s sarcoma. ③ Solitary Fibrous Tumor: It may exhibit mesenchymal hyalinization and collagenization, presenting with an angioendothelioma- like arrangement, necessitating differentiation from angioendothelioma. However, solitary fibrous tumors are rarely found in the subcutaneous tissue and typically exhibit dilated branching and antler-like vessels with significant surrounding hyalinization. Molecular genetics reveal *NAB2-STAT6* gene rearrangements; the STAT6-negative immunohistochemistry in this case does not support this tumor diagnosis. ④ Papillary endothelial hyperplasia(PEH): The lesion commonly occurs on the head and neck, fingers, and trunk, typically measuring less than 2 cm in diameter. It may display anastomosing vascular lumens and boot - shaped endothelial cells. The primary characteristic is the pseudopapillary or anastomosing growth of endothelial cells within intravascular thrombi. This case shows no significant intravascular growth, thus ruling out the diagnosis.

At initial diagnosis, the physician considered the morphology more consistent with a well-differentiated angiosarcoma. However, the patient was relatively young and had a prolonged disease course. The patient reported that the mass ceased growing after reaching a certain size, which is inconsistent with the rapid growth pattern typical of angiosarcoma. Additionally, the tumor exhibited mostly well-defined borders with parallel, arcuate, expansile infiltration patterns, lacking the cutting, invasive growth characteristics typical of well-differentiated angiosarcoma. There was no clear tumor necrosis, further inconsistent with angiosarcoma morphology. However, such highly active proliferation is uncommon in benign vascular tumors of adults. Further inquiry revealed the patient had been massaging the mass daily with medicated alcohol (specific details unknown) since its discovery, but with poor results, prompting hospital consultation. This suggests the high proliferation activity may stem from massage-induced stimulation of the mass. Of course, there is currently no established biological mechanism to support this hypothesis. It is worth noting that no recurrence cases were reported in the follow-up of such tumors in the literature. The case reported herein recurred after 10 months of postoperative follow-up, consistent with the tumor’s locally expansive, infiltrative growth pattern and its active proliferative cellular components. The case reported here recurred 10 months after surgery, which is consistent with the tumor’s locally expansive and infiltrative growth pattern, as well as its highly proliferative cellular composition; of course, the possibility that the surgical margins did not achieve R0 cannot be entirely ruled out. Since no preoperative imaging studies were performed to assess the extent of the tumor, although the resected mass appeared to have an intact capsule macroscopically and microscopically, tumor cells were found growing within the local capsule. Therefore, the possibility of tumor extrusion beyond the capsule cannot be ruled out, meaning it is uncertain whether the surgical margin was R0 or R1. This represents a limitation of this study.

## Conclusion

AH is a benign tumor for which there is no universally accepted standard treatment [10], but complete surgical resection remains the primary therapeutic approach. All currently reported cases exhibit no or low-grade atypia, with few mitotic figures and no recurrence. In this study, however, the case of AH demonstrated moderate atypia, numerous mitotic figures, and postoperative recurrence. Therefore, enhanced postoperative follow-up is warranted for cases with high proliferative activity. Due to morphological overlap with highly differentiated angiosarcomas and other entities, when differential diagnosis proves challenging, a comprehensive approach incorporating histology, immunohistochemistry, and molecular pathology is essential. Consultation with a soft tissue pathology specialist should be sought when necessary to prevent misdiagnosis and inappropriate treatment.

## Data Availability

The original contributions presented in the study are included in the article/supplementary material, further inquiries can be directed to the corresponding authors.
